# Editorial: Controversies and expectations for prevention and treatment of graft-versus-host-disease: a biological and clinical perspective

**DOI:** 10.3389/fimmu.2023.1212756

**Published:** 2023-05-15

**Authors:** Jacopo Mariotti, Daniel H. Fowler, Stefania Bramanti, Steve Z. Pavletic

**Affiliations:** ^1^ Bone Marrow Transplantation (BMT) and Cell Therapy Unit, IRCCS Humanitas Research Hospital, Rozzano, Italy; ^2^ Rapa Therapeutics, Rockville, MD, United States; ^3^ Immune Deficiency Cellular Therapy Program, Center for Cancer Research, National Cancer Institute, National Institutes of Health, Bethesda, MD, United States

**Keywords:** graft verses host disease, transplantation, allogeneic, combination (combined) therapy, review

Acute and chronic Graft-versus-Host-Disease (GVHD) remain the major contributors to transplantation related deaths and the most significant barrier to the success of allogeneic hematopoietic stem cell transplantation (allo-HSCT) (1). Despite prophylactic treatments with immunosuppressive agents, approximately 30-50% of transplantation recipients develop GVHD and, among them, at least 30% do not respond or relapse to front-line treatment and eventually die (2, 3). In this Research Topic, the authors have reviewed current options for prevention and treatment of GVHD and proposed new strategies to enhance available approaches in this field.

Watkins et al. provided a biological and clinical perspective of current platforms and future strategies for GVHD prophylaxis. The authors first summarized main risk factors contributing to GVHD onset and subsequently analyzed how current and new options for GVHD prophylaxis can affect them. The authors provided a detailed review of recent evidence on the employment of antithymocyte globulin (ATG) (4), post-transplant cyclophosphamide (PT-CY) (5) and anti CD80/CD86, abatacept (6), in the setting of matched related (MRD), matched (MUD) and mismatched (7/8) unrelated, and haploidentical donor transplants. Eventually they proposed personalized strategies based on different clinical situations, where we may need to optimize our GVHD preventive platform based on: underlying disease (high risk of relapse vs. non-malignant disease); risk of graft failure; or need of achieving rapid immune reconstitution to reduce infectious risks. Recent advances achieved with experimental strategies involving manipulation of the graft by CD45RA+ T cell depletion (7) or Treg cell enrichment (Orca-T) (8) are also discussed.

Several questions remain on the table: can we optimize GVHD prophylaxis by combining available immunosuppressive drugs or by building on existing platforms with manipulated T cell add-back? Can we identify whether it is better to employ one drug or another to avoid a particular worrisome subset of GVHD presentation? The articles by Weng et al. and Huang et al. has set the stage to answer these questions. Lung chronic GVHD (cGVHD) is one of the most life threatening side effects after allo-HSCT. Weng et al. described a significant reduction of bronchiolitis obliterans syndrome (BOS) in patients receiving haploidentical transplant (Haplo-SCT) and recognized that this effect is mainly mediated by ATG use. This finding is in agreement with one of the first observations on the role of ATG at diminishing cGVHD for MUD transplants whereby ATG infusion was associated with a significant reduction of post-transplant lung dysfunction (9). It is tempting to speculate that early post-transplant depletion of type 17 T cells (Th17) is one of the main mechanisms underpinning this beneficial late effect mediated by ATG, as this mechanism is thought to contribute to the recent impressive results achieved by the ROK2 inhibitor, belumosudil, for the treatment of cGVHD, particularly lung cGVHD (10). Huang et al. has also brought new life to basiliximab, a well-known anti-IL2 receptor monoclonal antibody, that had failed to improve the front-line treatment of acute GVHD (11). Huang et al. described a very low incidence of acute (aGVHD) and cGVHD (15% and 12%, respectively) by the combination of ATG and basiliximab. It is possible that an early usage of basiliximab eliminates highly replicating IL-2 dependent donor-specific alloreactive T cells immediately after Allo-HSCT, thereby paralleling one of the PT-Cy mechanistic effects. In a large retrospective EBMT study, the combination of ATG and PT-Cy in the setting of Haplo-SCT was tested by several authors and was recently associated with a lower incidence of cGVHD and a higher rate of engraftment compared with the use of PTCy alone (12). The work by Huang et al. underlined once again that using a lower dose of ATG (≤ 6 mg/kg) achieves an efficacious level of immunosuppression while sparing important side effects, such as infections (CMV) or disease relapse. In this sense, usage of lower doses of ATG was recently appropriately recommended by an international consensus panel (13).

Although treatment of aGVHD has recently improved after the introduction of ruxolitinb as standard therapy for the steroid refractory subset (14, 15), a great deal of work remains, as delineated by the contributions of Pan et al. and Bojanic et al. Specifically, Pan et al., through their T-cell transcriptomic studies, has identified extensive activation of glycolysis pathways in T cells before aGVHD onset. In particular, TCR, mTORC1, MYC, Hedgehog and Wnt/ß-catenin pathways were significantly enriched and may represent master regulators of metabolic reprogramming of T cells involved in aGVHD onset. These findings provide new potential targets to treat or prevent aGVHD. Glycolysis and metabolic reprogramming were shown to be of great relevance for T cell differentiation and persistence. For instance, GSK-3b/Wnt signaling is involved in T stem cell memory differentiation (T_SCM_) (16), which are not only relevant for long-lived anti-tumor effect but are also involved in acute (17) and chronic (18) GVHD development. Moreover, WNT pathway inhibitors were recently found to prevent experimental sclerodermatous cGVHD in pre-clinical models (19). Altogether, there is strong evidence supporting to test Wnt or glycolysis inhibitors as potential agents to prevent or treat GVHD in the near future. Finally, Bojanic et al. have extensively reviewed extracorporeal photopheresis (ECP) as an immunomodulatory treatment for cGVHD. By exploring T cell and biomarkers changes to highlight ECP response, the authors not only provide new tools to monitor or predict response to ECP in GVHD patients, but most importantly pave the way for new experimental approaches combining ECP with other immunomodulatory drugs. For instance, promising results were recently shown by combining ECP and ruxolitinib for the treatment of steroid refractory cGVHD, particularly BOS (20). Such combinations therapy holds promise as a future backbone of new strategies capable of eliminating problematic cases of GVHD. In another article of our Research Topic, Pinto et al. showed that Resolvin D1 represents an important gene involved in bone regeneration both by promoting osteoblast differentiation and reducing osteoclastogenesis. These mechanisms are not only important in the field of bone allograft regeneration, but may be of relevance in the setting of GVHD. Acute and chronic GVHD may target atypical organ sites such as the bone marrow (BM) niche (21), resulting in poor graft function and increased risk of death due to infection or hemorrhage. BM T-cell infiltration, endothelial damage and a reduced number of osteoblasts represent a hallmark of GVHD-mediated BM attack. Strategies capable of overcoming such GVHD side effects are relatively lacking, and currently only include TPO mimetics or atorvastatin. Pinto et al. provides a new potential target in order to treat T cell-mediated BM damage.

## Author contributions

JM performed literature research and wrote the manuscript, SB, DF, SP revised the manuscript. All authors contributed to the article and approved the submitted version.
